# Impact of integrated WASH and maternal and child health interventions on diarrhea disease prevalence in a resource-constrained setting in Kenya

**DOI:** 10.1186/s41182-024-00616-1

**Published:** 2024-08-30

**Authors:** Betty Muriithi, Ernest Apondi Wandera, Rie Takeuchi, Felix Mutunga, Cyrus Kathiiko, Mary Wachira, Joseph Tinkoi, Mirasine Meiguran, Pius Akumu, Valeria Ndege, Ryoichiro Mochizuki, Satoshi Kaneko, Kouichi Morita, Collins Ouma, Yoshio Ichinose

**Affiliations:** 1 Institute of Tropical Medicine, Nagasaki University-Kenya Medical Research Institute, P.O. Box 19993-00202, Nairobi, Kenya; 2 Centre for Virus Research, Kenya Medical Research Institute, Nairobi, Kenya; 3 Department of Public Health, International University of Health and Welfare, Otawara, Japan; 4 World Vision Kenya, Nairobi, Kenya; 5 World Vision Japan, Tokyo, Japan; 6 Department of Biomedical Sciences and Technology, Maseno University, Maseno, Kenya

**Keywords:** Diarrhea, Transformative WASH, Nutrition, Maternal and child health, Handwashing

## Abstract

**Background:**

Water, sanitation and hygiene (WASH) and child health interventions are proven simple and cost-effective strategies for preventing diarrhea and minimizing excess mortality. Individually, they are able to prevent diarrhea though sub-optimally, and their effectiveness when combined may be higher. This study examined the effect of integrated WASH and maternal and child health (MCH) interventions on prevalence of diarrhea, in a resource-limited setting in Kenya.

**Methods:**

A controlled intervention was implemented in Narok County. The interventions included WASH interventions integrated with promotion of MCH. A structured questionnaire was used to collect data on targeted indicators before and after the interventions. Data were analyzed using descriptive statistics and Chi-square to establish the impact of the interventions.

**Results:**

A total of 431and 424 households and 491 and 487 households in intervention and control sites, respectively, participated in the baseline and endline surveys. Following implementation of the interventions, prevalence of diarrhea decreased by 69.1% (95% CI: 49.6–87.1%) and 58.6% (95% CI: 26.6–82.4%) in the intervention and control site, respectively. Treatment of drinking water and animal husbandry practices were significantly associated with diarrhea post-interventions.

**Conclusions:**

Integrating WASH interventions with other diarrhea control strategies and contextualizing them to meet site-specific needs may effectively prevent diarrhea.

## Introduction

Diarrheal diseases bear important implications on child morbidity, mortality and future health. While they still remain an important cause of mortality among children [1, 2], morbidity due to diarrhea is of even greater concern. Diarrheal diseases have an estimated annual disability-adjusted life-years (DALYs) of more than 40 million among children younger than 5 years [3]. Further, frequent diarrhea affects a child’s growth and development and increases susceptibility to other infectious disease due to down regulation of the immune system [3], with downstream effects such as linear growth faltering and impaired cognitive development [4, 5], and potentially initiates a vicious cycle of infection and malnutrition [6].

Being water- and food-borne diseases with a fecal–oral route of transmission, diarrheal diseases have multiple exposure pathways that contribute to ingestion of pathogens [7], through complex mechanisms that include an interplay of fomites, vectors and reservoirs [8]. Hands and drinking water are considered important transmission pathways since they facilitate both direct and indirect exposure to pathogens. However, there is growing evidence that soils are highly contaminated with diarrheal pathogens, particularly of zoonotic origin [9, 10]. Ercumen et al., [11] reported that soil had the highest mean count of *E. coli* compared to water, hands, food and flies, while Kwong et al., [7] identified direct ingestion of soil as a primary pathway of ingesting *E. coli*, suggesting that soils are heavily contaminated with diarrhea pathogens, and may also act as reservoirs, driving continuous transmission.

Major fecal–oral routes are affected by water, sanitation and hygiene strategies that disrupt transmission along such routes. Formative research, however, highlights geophagy and transmission through fomites as critical transmission routes especially among children [12, 13], that not only bear high concentrations of pathogens, but also remain relatively unchanged by core WASH strategies [7]. Consequently, use of toilets and handwashing can prevent contamination of the environment with fecal pathogens or reduce ingestion of pathogens, but contamination by animal feces may be prevalent and continuously drive transmission. Additionally, adherence to good WASH practices is low in most vulnerable settings, which renders WASH interventions ineffective.

Current evidence therefore suggest that WASH interventions alone are not sufficient to disrupt transmission and minimize exposure to diarrheal pathogens [14, 15], since these do not traverse all transmission pathways. A systematic review [16] showed that WASH interventions reduce the risk of diarrhea infection by 27–53% among children aged below five years, suggesting substantial though suboptimal capacity to prevent diarrhea on their own. Exposure landscapes also differ widely and interventions that prove effective in one setting may not necessarily work in a different setting. Luby et al., [17] consequently reported that WASH interventions resulted to risk reduction of diarrhea infection in Bangladesh but failed to prevent infection in Kenya and Zimbabwe [18] in a controlled trial of combined diarrhea prevention interventions. Moreover, while it is clear that WASH interventions have capacity to lower exposure to diarrhea pathogens, it is evident that their implementation requires a comprehensive approach that is multidimensional in nature, encompassing not only transmission and exposure dynamics, but also societal constructs in order to sustain exposure prevention. Additionally, interventions must address infant-specific risk factors [19], particularly infant and young child health and feeding, and hygiene of child play spaces that traditional interventions have underexplored.

**Fig. 1:**
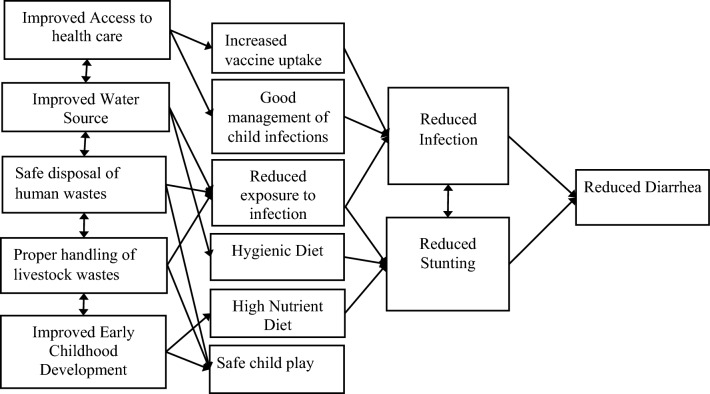
Conceptual framework of the project. The integrated water, hygiene and sanitation (WASH), maternal and child health (MCH), and child nutrition interventions would prevent exposure of children to diarrhea and improve their health and nutrition, with cumulative positive impact on prevention of childhood diarrhea

Considering exposure profile and dynamics of transmission of diarrhea diseases, the World Vision designed the Mother-to-Mother support project by combining diarrhea prevention strategies into an integrated and targeted WASH intervention, including water, sanitation and maternal and child health (MCH) with infant and child nutrition interventions in a resource-limited setting in Kenya. It was hypothesized that promoting antenatal care attendance, utilization of maternity services, childhood vaccination and child nutrition would improve health of children, reducing their susceptibility to infections, with WASH interventions enabling separation of persons from diarrhea pathogens and collectively disrupting transmission of diarrhea (Fig. 1). This study evaluated the impact of these interventions on the prevalence of diarrhea.

## Materials and methods

### Study site

The study was conducted in Narok South, Narok County, located in southwestern Kenya. The County lies within an arid and semi-arid region. Access to clean water is poor, coupled with relatively poor sanitation and hygiene conditions. Pastoralism is the main source of livelihood although subsistence crop farming is practiced in some areas. Health infrastructure is weak, with an average distance of more than 10 km between health facilities [20]. Utilization of health facilities particularly MCH services is low [21]. Nutritional status among children is rated medium with severe acute malnutrition [22] and stunting being the most prevalent forms of malnutrition [23, 24].

The project was implemented in Elangata-Enterit location while Maji-Moto location served as the control site. The intervention site was chosen because of its poor WASH conditions and poor child health rating. Maji-Moto was selected as the control site because its socio-economic status was similar to the intervention site. It is also geographically separated from the intervention site, which minimized control site contamination. The intervention site consisted of 11 villages spanning 3 sub-locations and is predominantly rural. The control site is located close to Narok town and had 9 villages. The area is predominantly rural with one semi-urban area. Communal land tenure was practiced in both sites although individual land tenure was later adopted in the control site during the intervention duration. Settlements were mostly temporary in the intervention site and semi-permanent in the control site, and land was largely used for livestock keeping. Each area is served by one public health facility.

### Study design

A controlled intervention study was conducted. A baseline survey was conducted in February 2018 in both sites to generate baseline information from which interventions were designed. All interventions were implemented from April 2018 to March 2021. An endline survey was thereafter conducted to evaluate the impact of the interventions.

### Study population

All households in Elangata-Enterit location were targeted by the interventions while all households in Maji-Moto location served as source population for the control arm. Study participants for both baseline and endline surveys were households with at least one child under the age of five years.

### Interventions

WASH interventions, including interventions for promotion of good animal husbandry practices combined with interventions aimed at improving MCH were implemented. The MCH interventions aimed to improve health seeking practices and increase utilization of health services, which would promote health of children from birth, while WASH interventions targeted household and environmental hygiene and sanitation.

A mega borehole was drilled at a central location in the intervention site and piped to surrounding schools, health facility and several water vending points in the community to improve access to clean water. The project also facilitated construction of toilets and handwashing structures in public spaces in collaboration with local authorities. The health facility at the intervention site was upgraded too. A maternity wing was constructed, together with a well-equipped laboratory. The outpatient clinic was also expanded to accommodate a MCH clinic.

The WASH interventions targeted domestic water quality, handwashing, treatment of drinking water, proper disposal of human feces and promotion of human–livestock separation. The MCH interventions aimed at increasing antenatal care attendance, delivery at the health facility, child vaccination and utilization of the health facility for management of childhood illnesses. These interventions were supplemented by a monthly mobile clinic in each location that offered immunization services, antenatal and postnatal check-ups and nutrition counselling.

The project further established structures for community education, consisting of community health volunteers (CHVs), three Citizen Voice and Action (CVA) groups and mother-to-mother support groups. The CHVs were recruited from within the community health strategy while the CVA groups consisted of local authority and key opinion leaders. The mother-to-mother support groups consisted of mothers and influential female figures in the community. Their role was to relay key messages at household and community levels on WASH, prenatal health, health facility delivery, child vaccination, exclusive breastfeeding and young child feeding, and good animal husbandry practices. These groups were all trained accordingly before roll-out of the interventions [25]. Three refresher trainings were conducted every project year to ensure that community education was conducted correctly. Continued community education focused on providing knowledge on when and how to perform targeted behaviors and practices, towards stimulating and sustaining habitual performance of desired practices.

### Sampling

Sample size was calculated based on the double population formula, at 95% CI, assuming a power of 80%, a ratio of unexposed to exposed of 1:1, and percent of outcome in unexposed of 50% and 65% in exposed group, to obtain the minimum sample. Given the scope of the interventions and the relatively large source population, a final sample size of 431 and 424 households and 491 and 487 households in the intervention and control site, at baseline and endline surveys, respectively, was reached. Sampling was done using proportionate sampling. There being a finite number of households in each village in both sites, villages were used as a baseline cluster to allocate the number of households to be sampled in every village. All villages were eligible to participate in the survey since they all consumed the interventions or all acted as controls. Within the villages, households were selected randomly. A household was required to be having at least one child under the age of five years in order to be included in the study.

### Data collection

A structured questionnaire that was developed based on outputs of the project and desired outcomes was used to collect data. Respondents were any adult member of a selected household. The questionnaire examined occurrence of diarrhea, demographic characteristics, MCH practices, childhood nutrition status and WASH practices. Occurrence of diarrhea at household level was established by asking whether the youngest child had experienced diarrhea in the two weeks prior to each survey. MCH practices were established by examining ANC attendance, place of delivery, breastfeeding practices, awareness and uptake of rotavirus vaccine, and attendance to postnatal care. Child’s mid upper-arm circumference was also measured, by specially trained enumerators using standard anthropometric tools to establish nutrition status. Source of water for the household in both rainy and dry season, water treatment, disposal of human feces, animal husbandry practices and handwashing were examined to establish WASH practices before and after interventions. Evaluation of handwashing focused on handwashing before eating and handling food only. Toilet coverage and usage was very low at the start of the interventions, coupled with social norms surrounding handling of human feces that were projected to affect outcomes. Examining handwashing after toilet usage was therefore excluded to minimize social desirability bias, especially after the interventions.

### Data analysis

Data were analyzed using STATA 14. Descriptive statistics were used to describe samples, establish distributions and calculate prevalence of diarrhea. Chi-square test was used to establish association between intervention cluster indicator and the outcome variable post-interventions, to establish effectiveness of the intervention. For all analyses, *p* < 0.05 was considered statistically significant.

## Results

### Demographic characteristics of study participants

A total of 431and 424 households and 491 and 487 households in the intervention and control sites, respectively, participated in the baseline and endline surveys. Majority of the respondents in both surveys were female, who had mostly never attended school. Mean household size increased from 3.5 (1–10) to 5.3 (2–12) in the intervention site and from 3.3 (1–9) to 6.1 (2–14) in the control site from baseline period to endline survey. Number of malnourished children in the intervention site decreased from 25 (6.6%) to 9 (2.2%) after the interventions. Decrease in the number of malnourished children was higher in the intervention site (Table 1).

**Table 1:** Household characteristics in the intervention and control sites, before and after the interventions

Variable	Intervention		Control	
	Baseline *n* = 431 (%)	Endline *n* = 491 (%)	Baseline *n* = 424 (%)	Endline *n* = 478 (%)
Gender of respondents				
Male	11 (2.5)	6 (1.2)	18 (4.3)	3 (0.6)
Female	420 (97.5)	485 (98.8)	406 (95.7)	484 (99.4)
Mean age	32.8 (19–78 years)	29.3 (16–56 years)	32.8 (19–81 years)	27 (15–50 years)
Highest level of education attended				
Never attended school	388 (90.0)	422 (85.9)	337 (79.5)	327 (67.2)
Primary school	38 (8.8)	29 (5.9)	71 (16.7)	29 (5.6)
Secondary and above	5 (1.2)	40 (8.1)	16 (3.8)	131 (27.9)
Mean number of household members (range)	3.5 (1–10)	5.3 (2–12)	3.3 (1–9)	6.1 (2–14)
Age of children*				
0–11	103 (27.4)	138 (28.2)	50 (11.4)	139 (28.7)
12–23	43 (11.4)	128 (26.1)	72 (16.4)	117 (24.1)
24–35	74 (19.7)	121 (24.6)	115 (26.5)	93 (19.2)
36 and above	156 (41.5)	104 (21.2)	202 (46.0)	136 (28.0)
Source of livelihood				
Livestock keeping	395 (91.6)	453 (92.3)	356 (84.0)	368 (75.6)
Crop growing	9 (2.1)	30 (6.1)	24 (5.7)	64 (13.1)
Employed	26 (6.0)	6 (1.2)	42 (9.9)	44 (9.0)
Middle upper arm circumference**				
Malnourished	25 (6.6)	9 (2.2)	15 (3.5)	14 (3.4)
At risk of malnutrition	74 (19.7)	52 (12.4)	50 (11.4)	36 (8.8)
Normal MUAC	277 (73.7)	357 (85.4)	374 (85.2)	361 (87.8)

*n of number of children was 376 in the intervention site and 439 in the control site at baseline

**n of MUAC during endline survey was 418 in the intervention site and 411 in control site

### Baseline and endline characteristics of the control and intervention sites

Access to improved water sources before the interventions was low, especially in the intervention site. Households that treated drinking water were also few before the interventions, increasing from 36.7% to 47.8% of the households in the intervention site. A decrease in the number of households that treated drinking water was observed in the control site after the interventions. Households in which all children had completed rotavirus vaccination increased in the intervention site relative to the control site post-interventions, while optimal attendance to ANC increased in the intervention site and decreased in the control site. Usage of a toilet for disposal of human feces was low, especially in the intervention site, and increased modestly post-interventions. Handwashing before eating and before preparing food decreased in both sites after the interventions (Table 2).

**Table 2:** Baseline and endline characteristics of the control and intervention sites

Characteristic	Intervention site		Control site	
	Baseline *n* = 431 (%)	Endline *n* = 491 (%)	Baseline *n* = 424 (%)	Endline *n* = 487 (%)
Households that utilized improved water sources during the dry season	37 (8.6)	175 (35.6)	113 (26.6)	136 (27.9)
Households that utilized improved water sources during the rainy season	109 (25.3)	418 (86.2)	156 (36.8)	425 (87.6)
Households that practiced treatment of drinking water	158 (36.7)	64 (47.8)	198 (46.7)	25 (41.0)
Households that used a toilet for disposal of human feces	6 (1.4)	45 (9.2)	81 (19.1)	143 (29.4)
Households that disposed a child's recent stool safely	15 (3.7)	94 (19.4)	58 (13.9)	126 (25.9)
Participants who perceived open defecation as a risk for diseases in the community	356 (82.6)	310 (63.1)	380 (89.6)	384 (78.8)
Households that housed livestock in dwelling house	122 (28.3)	155 (32.1)	47 (11.1)	75 (17.8)
Households that housed livestock in a fenced shed within the home	69 (16.0)	461 (95.5)	22 (5.2)	390 (92.4)
Households that housed livestock in free range	316 (73.3)	43 (8.9)	356 (84.0)	37 (8.8)
Respondents who washed their hands before eating	412 (95.6)	409 (83.3)	415 (97.8)	436 (89.5)
Respondents who washed their hands before preparing food	387 (89.8)	415 (84.5)	396 (93.4)	398 (81.7)
Respondents who delivered youngest child in a health facility	19 (4.6)	238 (48.7)	40 (9.5)	257 (53.1)
Respondents who were aware of the rotavirus vaccine	273 (63.3)	361 (73.5)	341 (80.4)	320 (65.7)
Children who had completed rotavirus vaccination	165 (38.3)	324 (89.7)	204 (48.1)	219 (68.9)
Children with incomplete rotavirus vaccination	88 (20.4)	18 (5.0)	118 (27.8)	73 (23.0)
Respondents who attended more than 4 ANCs in last pregnancy	208 (48.3)	295 (60.1)	295 (69.6)	273 (55.9)
Respondents who attended PNC after last pregnancy	272 (63.1)	287 (58.5)	397 (93.6)	190 (39.2)
Children who were exclusively breastfed	278 (64.5)	376 (77.7)	121 (28.5)	406 (84.4)

### Prevalence of diarrhea at baseline and endline surveys

**Fig. 2:**
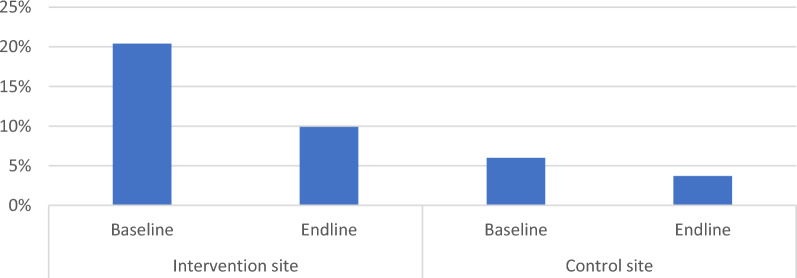
Prevalence of diarrhea before interventions was 20.4% (*n* = 431) and 9.9% (*n* = 424) in the intervention and control sites, respectively. After the interventions, prevalence decreased to 6.3% (*n* = 491) and 4.1% (*n* = 487) in the intervention and control sites, respectively

Prevalence of diarrhea over two weeks preceding the baseline survey was higher in the intervention site compared to the control site (Fig. 2). Prevalence decreased from 20.4% (95% CI: 16.7–25.5%) to 6.3% (95% CI:4.3–8.8%) and from 9.9% (95% CI: 7.2–13.2%) to 4.1% (95% CI: 2.5–6.3%) in the intervention and control sites, respectively, after the interventions. This represented a diarrhea disease decrease of 69.1% (95% CI: 49.6–87.1%) and 58.6% (95% CI: 26.6–82.4%) in the intervention and control sites, respectively.

### Impact of the interventions on diarrhea

After the interventions, 47.8% of the households in the intervention site were practicing treatment of drinking water and treatment of drinking water was significantly associated with occurrence of diarrhea (*p* = 0.034). More households (95.5%) in the intervention site were housing their livestock in a secluded shed within the home compound with much less (8.9%) practicing free-range husbandry, and these were significantly associated with occurrence of diarrhea as shown on Table 3 (*p* = 0.001 and *p* =  < 0.001, respectively). Increase in attendance to ANC and PNC was higher in the intervention site compared to control site although they were not significantly associated with occurrence of diarrhea.

**Table 3:** Impact of the interventions on diarrhea

Characteristic	Category	Intervention *n* = 491 (%)	*p*-value	Control *n* = 487 (%)	*p*-value
Dry season water source	Improved	175 (35.6)	0.684	136 (27.9)	0.072
	Unimproved	316 (64.4)		351 (72.1)	
Rainy season water source	Improved	418 (86.2)	0.355	425 (87.6)	0.307
	Unimproved	67 (13.8)		60 (12.4)	
Treatment of drinking water	Treated water	64 (47.8)	0.034	25 (41.0)	0.226
	Did not treat water	70 (52.2)		36 (59.0)	
Disposal of human feces	Used a toilet	45 (9.2)	0.165	143 (29.4)	0.292
	Didn't use a toilet	446 (90.8)		344 (70.6)	
Disposal of a child’s stool	Safe	94 (19.4)	0.659	126 (25.9)	0.012
	Unsafe	397 (80.9)		361 (74.1)	
Perception of open defecation as a risk for diseases	Risk	310 (63.1)	0.264	384 (78.8)	0.02
	Not a risk	8 (1.6)		18 (3.7)	
	Don’t know	173 (35.2)		85 (17.5)	
Housing livestock	In dwelling	155 (32.1)	0.579	75 (17.8)	0.526
	In a fenced shed	461 (95.5)	0.001	390 (92.4)	0.228
	Free range	43 (8.9)	< 0.001	37 (8.8)	0.668
Handwashing before eating	Yes	409 (83.3)	0.057	436 (89.5)	0.412
	No	82 (16.7)		51 (10.5)	
Handwashing before preparing food	Yes	415 (84.5)	0.682	398 (81.7)	0.433
	No	76 (15.5)		89 (18.3)	
Place of delivery	Health facility	238 (48.7)	0.686	257 (53.1)	0.459
	Home	251 (51.3)		227 (46.9)	
Rotavirus vaccine awareness	Aware	361 (73.5)	0.177	320 (65.7)	0.67
	Not aware	130 (26.5)		167 (34.3)	
Rotavirus vaccination	All children completed	324 (89.7)	0.366	219 (68.9)	0.703
	Not all children completed	18 (5.0)		73 (23.0)	
	No child vaccinated	11 (3.0)		24 (7.5)	
ANC attendance	≥ 4 ANC visits	295 (60.1)	0.948	273 (55.9)	0.476
	< 4 ANC visits	169 (34.4)		192 (39.6)	
	Never attended ANC	27 (5.5)		22 (4.5)	
Attendance to PNC	Attended PNC	287 (58.5)	0.673	190 (39.2)	0.391
	Did not attend PNC	204 (41.55)		295 (60.8)	
Breastfeeding status of youngest child	Exclusive	376 (77.7)	0.753	406 (84.4)	0.941
	Supplemented	108 (22.3)		75 (15.6)	

## Discussion

This study implemented an integrated WASH and MCH intervention in a low-income setting, incorporating interventions for provision of safe water for domestic use, household and environmental sanitation and hygiene, and improved antenatal and postnatal care for mothers and children. The interventions successfully improved WASH and MCH indicators in the intervention site. Prevalence of diarrhea decreased by 69.1% and 58.6% in the intervention and control sites, respectively, after the interventions. Water treatment and good animal husbandry practices were significantly associated with occurrence of diarrhea in the intervention site post-interventions.

More households in the intervention site treated drinking water, compared to the control site after the intervention period. The mother-to-mother project undertook continued sensitization of the community on the importance of water treatment at both household and community levels, that possibly prompted more households to treat drinking water. Moreover, Wandera et al., [26] observed fecal coliforms in water sampled at the point of use even after improvement of water sources implying persistence of microbial contaminants in domestic water. Treating water for drinking has been shown to significantly lower the risk of diarrhea [14, 27], and capacity of various water treatment methods to eliminate pathogens from drinking water has been demonstrated [28, 29]. Drinking water may therefore be a major diarrhea transmission pathway in this population, and water treatment may be an effective intervention for preventing diarrhea. Consequently, increased access to improved water source during both rainy and dry seasons was not significantly associated with diarrhea, suggesting that improving water sources alone may not be sufficient to prevent diarrhea in this setting.

The study further observed a substantial decrease in cohabitation with livestock and a shift from housing livestock in free-range within the home compound to housing them in a secluded enclosure within the home compound in the intervention site. Animal feces contaminates water and soil and are associated with increased risk for diarrhea and environmental enteropathy [11], with widespread contamination in settings where free-range animal husbandry practices are common. This being a pastoralist community, it is possible that soils and waters were substantially contaminated with pathogens from animal feces. Contamination by animal feces may have exceeded contamination by human feces, since usage of toilets for defecation and safe disposal of children’s feces were not significantly associated with diarrhea, despite considerable increase in access to toilets post-interventions. This finding implies that in settings where children’s likelihood of contact with and ingestion of soil is high, and where human–livestock contact is high, managing fecal contamination of the soil by livestock feces may deliver additional benefits over addressing contamination of the environment by human feces only.

The study did not observe desired improvements in handwashing practices, despite the critical role of hands in completing the diarrhea transmission chain. Handwashing interventions sought to promote handwashing before eating and before preparing food. Reported handwashing at targeted moments decreased after the intervention period in both sites, remaining slightly higher in the control site compared to the intervention site. Bias due to self-reported handwashing possibly contributed to high rates of handwashing before the interventions, as respondents may have given socially acceptable reports. Post-interventions, the study relied on the presence of a handwashing station as a proxy for handwashing, possibly resulting to observed low adherence to handwashing. Besides reporting bias, observed handwashing pattern could be due to perceived relative importance of washing hands, given water scarcity and low literacy characteristic of this population, or due to factors such as convenience in location of handwashing station [30]. Moreover, adherence to handwashing is extensively dependent on sustained behavior change that requires high inputs and intensive contact [18, 31] and preferably more intensive strategies of stimulating behavior change [32] that were not achievable within the scope of this study. Higher rates of handwashing in the control site was possibly due to unstructured improvements in socio-economic conditions.

Integrating WASH with MCH interventions in this study may have contributed to the observed decline of diarrhea at the end of the interventions. Targeted WASH and MCH indicators improved substantially, although only two interventions were prominently associated with diarrhea post-interventions. Generally, causes of diarrhea are multifaceted, implying the need for multisectoral and integrated approaches to address exposure. WASH interventions have therefore been proven capable of reducing exposure to diarrheal pathogens [33, 34] but have yielded mixed results in prevention of diarrhea [18, 31, 35]. Since combining interventions addresses multiple routes of transmission simultaneously, the interventions may have benefited from potential synergy achieved by implementing interventions in combination [36]. Targeting of interventions also possibly explains observed outcomes, since the interventions were specifically designed to address access to safe water for domestic use and poor child health ratings in a low-resource context with high human–livestock contact, as core exposures to diarrhea. Customizing the interventions to match prevailing conditions within the study population allowed for targeting of dominant transmission pathways that probably disrupted transmission. Unlike conventional WASH interventions, this study also targeted environmental contamination by livestock feces that have been shown to be an important source of environmental contaminants [11, 37], which was potentially high in the intervention site due to free-range livestock keeping practice. Given mouthing tendency of children, in a possibly highly contaminated environment, this study further focused on maintenance of play space hygiene to reduce exposure to pathogens, coupled with building child health through promoting vaccine uptake and child nutrition to increase immunity to infection. The interventions therefore not only disrupted transmission of diarrheal pathogens, but also potentially reduced fecal contamination in the environment, that collectively may have lowered occurrence of diarrhea.

Findings of this study contrast recent studies [17, 31, 38, 39], that showed that combining WASH interventions with nutritional interventions did not have significant protective effect against diarrhea. Those interventions possibly failed to reduce transmission of diarrhea pathogens due to high baseline prevalence of diarrhea [38], low intervention dose and adherence [31], coverage of intervention and the generally high fecal contamination in the environment [18]. Moreover, numerous studies have shown that exposure landscapes are diverse and a unique mix of interventions may be required in each setting or even season, which was probably not considered during design of these interventions. Our findings therefore suggest that a multidimensional targeted approach may be beneficial in design of WASH interventions, with careful consideration of prevailing WASH and exposure conditions.

This project had some limitations that might have influenced its results. First, private land tenure was adopted in the control site shortly after the project began that promoted permanent settlements, resulting to improvement of social and economic conditions. Improvement of some of the targeted outcomes was therefore observed in both sites substantially masking the true effect of the intervention. Secondly, the project utilized a non-randomized and observational approach that is susceptible to confounders and has little capacity of detecting small changes. Finally, outbreak of COVID-19 may have affected effective application of community education interventions following orders against social gatherings, during which messages on targeted behavior were passed. The COVID-19 restrictions also minimized household visits which substantially reduced contact between health educators and households, with a potential possibility of hindering stimulation of behavior change and hence uptake and adherence to some interventions.

## Conclusion

Treatment of drinking water alongside good animal husbandry practices potentially lowered exposure to diarrhea prevalence resulting to decreased prevalence of diarrhea post-interventions. These outcomes suggest that contextualizing diarrhea interventions to fit prevailing exposure conditions and implementing them in combination may effectively prevent transmission of diarrhea.

## Data Availability

Data and materials used to conduct this study can be available on request to the corresponding author.

## Abbreviations

WASH: Water, sanitation and hygiene
MNCH: Maternal, neonatal and child health
DALY: Disability-adjusted life year
KDHS: Kenya Demography and Health Survey
ECD: Early childhood development
CHVs: Community health volunteers
CVA: Citizen Voice and Action
IYCF: Infant and young child feeding
CHEWs: Community health extension workers
WHO: World Health Organization
COVID-19: Corona virus disease 2012
